# Efficacy and risks of fondaparinux 7.5 mg for deep vein thrombosis after total knee arthroplasty

**DOI:** 10.20407/fmj.2017-020

**Published:** 2018-12-06

**Authors:** Sho Nojiri, Kazue Hayakawa, Hideki Date, Harumoto Yamada

**Affiliations:** Department of Orthopedic Surgery, Fujita Health University School of Medicine, Toyoake, Aichi, Japan

**Keywords:** Deep vein thrombosis, Fondaparinux, Pulmonary thromboembolism, Total knee arthroplasty

## Abstract

**Objectives:**

High-dose fondaparinux therapy at 7.5 mg/day (FPX 7.5 mg) for deep vein thrombosis (DVT) may increase the risk of hemorrhage. We investigated the efficacy and safety of FPX 7.5 mg to treat DVT after total knee arthroplasty.

**Methods:**

This study included 101 patients (91 with osteoarthritis, 10 with rheumatoid arthritis; mean age at total knee arthroplasty: 72.9 years) with asymptomatic postoperative DVT. Medical prophylaxis for DVT was started on postoperative day 1. Vascular ultrasound was conducted within 2 days postoperatively; patients were switched to FPX 7.5 mg after DVT diagnosis. Ultrasound was repeated to monitor DVT resolution. Adverse reactions were assessed.

**Results:**

DVT resolved in 72 patients (71.3%) receiving FPX 7.5 mg. There were no significant differences between patients with versus without DVT resolution in the timing of FPX 7.5 mg therapy, treatment period, age, body mass index, or D-dimer or hemoglobin levels. There was no significant difference in DVT outcome between patients starting FPX 7.5 mg within 4 days postoperatively versus on day 5 or later, or between patients treated for ≤7 versus ≥8 days. Hemoglobin decreased to ≤7 g/dL in three patients (2.9%).

**Conclusions:**

FPX 7.5 mg can be expected to resolve DVT in 71.3% of patients; however, the risk of associated hemorrhagic complications may be higher than the risk of pulmonary embolism. To treat DVT with FPX 7.5 mg without compromising safety, patients should be selected carefully and the timing of treatment should be adjusted appropriately.

## Introduction

Although fondaparinux (FPX) is commonly used at doses of 1.5 or 2.5 mg/day to prevent deep vein thrombosis (DVT), our literature search did not find any articles on the use of FPX at a dose of 7.5 mg/day (FPX 7.5 mg) for the treatment of DVT after total knee arthroplasty (TKA). We investigated the efficacy and adverse effects of FPX 7.5 mg in the treatment of postoperative DVT, and we herein report our findings concerning the effective dosing regimen and complications.

DVT is a common and important postoperative complication after TKA. Because DVT of the lower extremity can lead to fatal pulmonary thromboembolism (PTE), several methods of prevention are necessary, such as early postoperative ambulation, physical therapy, and/or drug therapy. However, complete prevention is not possible and progression to PTE must be prevented in patients with fresh DVT. Anticoagulant therapy is expected to be more effective for fresh DVT than old DVT, and we employ early initiation of anticoagulant therapy to treat fresh DVT.

In recent years, there have been various advances in anticoagulant therapy for DVT. In addition to warfarin, which has long been used, FPX, which is an injectable synthetic factor Xa inhibitor, is now available, as are several novel oral anticoagulants.

Before performing TKA, we investigate the possible presence of DVT with vascular ultrasound of the lower extremity. Since January 2012, we have administered FPX 7.5 mg to patients in whom DVT was suspected or detected with vascular ultrasound. Although this treatment is expected to be effective, it may cause hemorrhage because 7.5 mg is three to five times the dose used for DVT prophylaxis (1.5 or 2.5 mg). DVT can be dissolved effectively if FPX treatment is started soon after surgery; however, the medication increases the risk of postoperative hemorrhage. Therefore, we need to consider safety when determining the appropriate anticoagulant regimen.

## Methods

From January 2012 to December 2015, 323 patients underwent vascular ultrasound both before and after TKA and were confirmed to have no DVT preoperatively. Among these, 101 patients (18 men and 83 women) were included in this retrospective study. All 101 patients had DVT confirmed with postoperative vascular ultrasound and were treated with FPX 7.5 mg to dissolve the thrombus. The average age of patients was 72.9 years (59–89 years). The underlying disease was osteoarthritis of the knee in 91 patients and rheumatoid arthritis in 10 patients. TKA was bilateral in 35 patients and unilateral in 66. DVT was evaluated with vascular ultrasound of the lower extremity (Aplio 500; Toshiba Medical Co.) using a linear probe with a central frequency of 7.5 MHz. The common femoral vein, superficial femoral vein, and popliteal vein were imaged, as were the distal veins (posterior tibial, peroneal, gastrocnemius, and soleus veins). For evaluation of the central veins, respiratory fluctuation or milking was adopted as required. For evaluation of DVT, the probe was used to compress the vessel and resulting changes in the lumen were assessed. Lack of change in the lumen was defined as positive for DVT. DVT of the leg veins was detected at a total of 109 locations, including in the soleus vein in 86 patients (79%), the gastrocnemius vein in 13 patients (12%), the posterior tibial vein in three patients (3%), and the peroneal vein in seven patients (6%). DVT was asymptomatic in all patients; no proximal DVT was detected.

According to our protocol, anticoagulant therapy with enoxaparin (2000 IU b.i.d.) or edoxaban (30 mg/day) was started for DVT prophylaxis on postoperative day 1 after TKA. Vascular ultrasound was conducted on day 1 or 2 after TKA; patients with confirmed or suspected DVT were switched to FPX 7.5 mg. This study was conducted retrospectively; therefore, the timing of examination and subsequent FPX initiation varied among patients because of their condition or scheduling difficulties. Treatment with FPX 7.5 mg was started 2–10 days after TKA (mean: 5.1 days); the treatment period was 3–20 days (mean: 7.5 days). Ultrasound examinations were repeated in weeks 1 and 2 after TKA to confirm DVT dissolution. Transfusion of autologous blood or packed red cells was performed from immediately postoperatively to 2 days after surgery, as required for each patient.

We investigated the treatment effect of FPX 7.5 mg (resolution or persistence of DVT, confirmed with vascular ultrasound) and compared various factors between patients with DVT resolution versus those in whom thrombus persisted (age, sex, body mass index [BMI], underlying disease, timing of treatment initiation, duration of treatment, and D-dimer level). In addition, we compared patients who started FPX treatment ≤4 days after surgery versus those who started treatment ≥5 days after surgery, as well as patients who received FPX for ≤7 days versus those treated for ≥8 days. Changes in hemoglobin (Hb) and the occurrence of major bleeding were also compared between patients with DVT resolution versus those with persistent DVT.

The Mann–Whitney U test was used to compare the timing of treatment initiation, duration of treatment, age, BMI, change in D-dimer, and change in Hb in relation to the resolution or persistence of DVT. Fisher’s exact test was used to assess the influence of sex, underlying disease, initiation of treatment on day 4 or earlier versus day 5 or later, and treatment duration of ≤7 days versus ≥8 days.

## Results

Vascular ultrasound confirmed DVT resolution in 72 patients and persistence in 29 patients (DVT resolution rate: 71.3%). Resolution rates according to location were 57% (49/86) for the soleus vein, 77% (10/13) for the gastrocnemius vein, 100% (3/3) for the posterior tibial vein, and 85% (6/7) for the peroneal vein.

There were no significant differences between patients with versus without DVT resolution in the timing of treatment initiation, duration of treatment, age, BMI, change in D-dimer, or change in Hb (Table 1).

There were also no significant differences between patients with versus without DVT resolution in sex ratio or underlying disease (Table 2). The influence of the timing of FPX 7.5 mg initiation was assessed by comparing patients who started treatment for DVT ≤4 days after surgery versus ≥5 days after surgery; no significant difference in DVT resolution was observed between these groups (Table 2). Similarly, DVT resolution rates were compared between patients treated with FPX 7.5 mg for ≤7 days versus ≥8 days; no significant difference was observed between these groups (Table 2).

There was no significant difference in Hb between patients with versus without DVT resolution. In three patients (2.9%), Hb decreased to ≤7.0 g/dL on postoperative day 3 or later; FPX 7.5 mg was initiated on postoperative day 3 in all three of these patients. Two of these patients received additional transfusion; FPX 7.5 mg was discontinued in one patient (Table 3). There were no surgical complications of TKA, including infection, repeat surgery, or hemorrhage requiring additional treatment. Although major or clinically significant perioperative bleeding was not observed in these three patients, the two patients who received additional blood transfusion were classified as having clinically significant bleeding after consideration of their postoperative course. There were no episodes of major bleeding or symptomatic PTE. Screening for asymptomatic PTE was not performed.

## Discussion

In TKA patients who develop PTE, the mortality rate is high (11.9%–22%), despite early treatment^1,2^; 40% of deaths resulting from PTE occur within 1 hour of onset.^1^ In Japan, the reported incidence of perioperative PTE is 0.076% among patients undergoing hip or lower limb surgery.^3^ The reported incidence of PTE associated with total hip arthroplasty and TKA is 0.55%, with significant differences depending on the use of prophylaxis.^4^

TKA is a highly thrombogenic operation,^5–10^ with thrombus detected in 16.7%–42% of TKA patients who undergo vascular ultrasound.^5,6,8–10^ Although the incidence of thrombus varies according to the regimen used for DVT prophylaxis, the reported ranges are 24%–34% for distal thrombus, 2%–11% for proximal thrombus, and 0%–0.6% for PTE.^5^ The reported incidence of symptomatic DVT on day 10 after surgery is 0.9%.^6^

In a study that stratified patients according to DVT risk, mechanical prophylaxis for DVT was shown to be safe and useful after TKA^11^; the incidence of DVT was reported to be 6.6% with mechanical prophylaxis alone.^12^ However, other studies found a much higher DVT incidence of 16.2%–29.8% from 10 to 14 days after TKA, despite prophylactic therapy,^13–15^ suggesting that thrombosis cannot be avoided in some patients.

Patients undergo TKA with the hope of improving their level of activity. Therefore, fatal complications should be prevented by all means possible, including proactive prophylaxis and treatment of DVT. For early detection of DVT in patients without preoperative thrombus, we perform vascular ultrasound at 1–2 days, 1 week, and 2 weeks after surgery. If DVT is detected postoperatively, it is classified as fresh DVT and treated with FPX 7.5 mg.

FPX 7.5 mg was approved in Japan in January 2011; however, there have been few reports on its use for the treatment of acute DVT and acute PTE. In the MATISSE-PE study^16^ conducted by Buller et al., 2213 patients with acute symptomatic PTE were randomized to receive treatment with FPX (5 mg, 7.5 mg, or 10 mg) or unfractionated heparin. Major bleeding occurred in 14 of 1092 patients (1.3%) who received FPX. It has also been reported that FPX is useful for patients with heparin-induced thrombocytopenia.^17^

In the present study, the timing of treatment initiation, duration of treatment, age, BMI, D-dimer level, and Hb level were not significantly associated with the efficacy of FPX 7.5 for DVT, making it difficult to predict the efficacy of this drug. While there was no significant association between efficacy for DVT resolution and Hb level, FPX 7.5 mg was started on postoperative day 3 in all three patients whose Hb decreased to ≤7 g/dL. In general, we found that Hb declined for 5 days after TKA (Table 1). If thrombus is detected with vascular ultrasound within 2 days postoperatively, we consider it safer to continue medical prophylaxis for DVT until postoperative day 5 and then start FPX 7.5 mg on that day.

We found no significant difference in outcomes between patients treated with FPX 7.5 mg for ≤7 days versus those treated for ≥8 days. However, DVT persisted in 20 patients who received treatment for ≤7 days and resolved in 22 patients treated for 8–20 days (mean: 12.3 days). These results suggest that DVT might have resolved in more of the patients treated for ≤7 days if they had continued treatment for longer (Table 2).

Our study had a retrospective design. To further elucidate the efficacy of FPX, an intervention study is needed that compares results stratified according to the timing of administration initiation, treatment period, and presence/absence of FPX use, as well as an evaluation of the prevention of PTE and the incidence of complications.

Vascular ultrasound achieves a high detection rate of DVT after TKA^5,6,8–10^ and the thrombus is usually distal. In the present study, all patients treated with FPX 7.5 mg had acute DVT that was asymptomatic and distal. There have been few reports concerning the management of distal DVT. Currently, the Clinical Practice Guidelines of the American Academy of Orthopaedic Surgeons and the 2012 American College of Chest Physicians Guidelines do not recommend venous ultrasound screening or proactive treatment of asymptomatic isolated distal DVT.^18^ According to the American College of Chest Physicians Guideline of 2016,^19^ diagnostic imaging every 2 weeks is recommended for DVT limited to the lower extremity. If thrombi show extension following surgery, anticoagulant therapy is recommended; however, screening is not recommended. The duration of hospitalization following TKA has shortened in recent years, and biweekly examinations are expected to be difficult in practice. Although the Japanese Orthopedic Association stated in its 2017 Guideline for the Prevention of Symptomatic Deep Vein Thrombosis^20^ that routine screening with vascular ultrasound for asymptomatic DVT following TKA is not recommended, the guideline also stressed that superficial or slavish adherence to the guideline should be avoided. In this study, we performed vascular ultrasound before and after surgery in all patients undergoing TKA. Because of costs, it would be difficult to continue performing ultrasound assessment and pharmacological prophylaxis in all patients. Doctors also need to strike a balance between the risks of thrombosis and hemorrhage. At present, we are determining the necessity for testing on a patient-by-patient basis.

Hb decreased in three patients (2.9%) in the present study. This percentage is higher than the incidence of PTE,^3,4^ suggesting that the risk of bleeding with FPX 7.5 mg may exceed the risk of PTE without it. Large-scale clinical studies such as HOKUSAI^21^ have demonstrated that direct oral anticoagulants are not inferior to warfarin in safety or efficacy, and that they have a significantly lower risk of hemorrhage. When considering FPX 7.5 mg, patients should be selected carefully and use should be avoided in those with asymptomatic distal DVT.

The chief limitation of this study was that it only assessed treatment with FPX 7.5 mg and there was no control group. However, FPX 7.5 mg was used to treat all patients with fresh DVT after surgery, and it would have been ethically problematic to offer suboptimal treatment to a control group.

In addition, the relationship between blood transfusion and DVT was not investigated, nor was the long-term incidence of PTE. However, we do not think that blood transfusion significantly influenced the onset of DVT. Regarding the detection of PTE, contrast-enhanced chest CT was not performed because our patients did not have ventilatory impairment or dyspnea.

We investigated the efficacy and safety of FPX 7.5 mg in 101 patients with DVT detected with vascular ultrasound following TKA. The DVT resolution rate was 71.3%. There were no significant differences in the timing of treatment, duration of treatment, age, or D-dimer level between patients with versus without resolution of DVT. Our findings regarding Hb suggest that initiation of treatment with FPX 7.5 mg on postoperative day 5 or later may be effective. Hb decreased to ≤7 g/dL in three patients (2.9%) who started FPX 7.5 mg on postoperative day 3, suggesting that the risk of bleeding may exceed the risk of PTE with early initiation of treatment.

FPX 7.5 mg appears to be safe and highly effective for DVT if administration is limited to carefully selected patients and if timing of treatment is adjusted appropriately.

## Figures and Tables

**Table1:** Patient characteristics according to resolution versus persistence of DVT

	Patients with DVT persistence (n=29)	Patients with DVT resolution (n=72)	P value*
Treatment initiation, POD	4.9±3.0	5.7±2.5	0.38
Treatment duration, days	7.5±3.8	7.2±4.1	0.17
Age, years	74.6±4.6	72.3±6.7	0.15
BMI, kg/m^2^	25.9±3.0	25.1±3.5	0.25
D-dimer level, units 6 hours after surgery	43.7±74.5	39.9±38.4	0.43
Day 1 after surgery	28.5±26.7	29.1±23.9	0.44
Day 2 after surgery	10.2±8.9	8.7±8.0	0.36
Day 5 after surgery	13.3±8.6	11.3±6.1	0.51
Day 7 after surgery	15.6±10.4	13.7±7.3	0.78
Day 14 after surgery	15.8±12.5	14.6±8.6	0.85
Day 21 after surgery	15.1±11.9	13.5±7.6	0.67
Hb, g/dL 6 hours after surgery	10.6±2.0	11.2±1.5	0.14
Day 1 after surgery	10.8±1.5	11.4±1.5	0.12
Day 2 after surgery	10±1.2	10.4±1.5	0.11
Day 5 after surgery	9.6±1.4	9.9±1.5	0.74
Day 7 after surgery	10±1.5	10.1±1.4	0.95
Day 14 after surgery	10.5±1.0	10.6±1.3	0.98
Day 21 after surgery	10.8±0.9	10.9±1.2	0.79

BMI=body mass index, DVT=deep vein thrombosis, POD=postoperative day *Mann–Whitney U test

**Table2:** Resolution of DVT according to sex, underlying disease, timing of treatment initiation, and treatment duration

		DVT resolved (no. of patients)	DVT persisted (no. of patients)	
Sex	Male	14	4	N.S.*
	Female	58	25	
Underlying disease	Osteoarthritis	65	26	N.S.*
	Rheumatoid arthritis	7	3	
Timing of treatment initiation	Days 1 to 4 after surgery	33	13	N.S.*
	Day 5 or later	39	16	
Treatment duration	≤7 days	50	20	N.S.*
	8 to 20 days	22	9	

DVT=deep vein thrombosis, N.S.=not significant *Fisher’s exact test

**Table3:** Details of patients in whom Hb decreased to 7.0 g/dL or lower

	Age, years	Sex	FPX started	Hb on POD 5, g/dL	Additional blood transfusion	FPX continued	Resolution of DVT
Patient 1	59	Female	POD 3	6.7	No	Continued for 8 days	Resolved after 1 week
Patient 2	69	Female	POD 3	6.9	Yes	Discontinued	Resolved after 6 weeks
Patient 3	75	Male	POD 3	7	Yes	Continued for 7 days	Resolved after 1 week

FPX=fondaparinux, Hb=hemoglobin, POD=postoperative day
